# Mammary inflammation around parturition appeared to be attenuated by consumption of fish oil rich in n-3 polyunsaturated fatty acids

**DOI:** 10.1186/1476-511X-12-190

**Published:** 2013-12-31

**Authors:** Sen Lin, Jia Hou, Fang Xiang, Xiaoling Zhang, Lianqiang Che, Yan Lin, Shengyu Xu, Gang Tian, Qiufeng Zeng, Bing Yu, Keying Zhang, Daiwen Chen, De Wu, Zhengfeng Fang

**Affiliations:** 1 Key Laboratory for Animal Disease Resistance Nutrition of the Ministry of Education of China, Animal Nutrition Institute, Sichuan Agricultural University, Sichuan, Ya’an 625014, China

**Keywords:** Mastitis, n-3 PUFA, Inflammatory cytokines, IL-10, PPAR-γ

## Abstract

**Background:**

Mastitis endangers the health of domestic animals and humans, and may cause problems concerning food safety. It is documented that n-3 polyunsaturated fatty acids (PUFA) play significant roles in attenuating saturated fatty acids (SFA)-induced inflammation. This study was therefore conducted to determine whether mammary inflammation could be affected by consumption of diets rich in n-3 PUFA.

**Methods:**

Forty-eight rats after mating began to receive diets supplemented with 5% fish oil (FO) or 7% soybean oil (SO). Blood and mammary tissue samples (n = 6) at day 0 and 14 of gestation and day 3 postpartum were collected 9 hours after intramammary infusion of saline or lipopolysaccharide (LPS) to determine free fatty acids (FFA) concentration and FA composition in plasma and inflammation mediators in mammary tissues.

**Results:**

At day 14 of gestation and day 3 postpartum, the FO-fed rats had lower plasma concentrations of C18:2n6, C20:4n6, total n-6 PUFA and SFA, and higher plasma concentrations of C20:5n3 and total n-3 PUFA than the SO-fed rats. Plasma C22:6n3 concentration was also higher in the FO-fed than in the SO-fed rats at day 3 postpartum. Compared with the SO-fed rats, the FO-fed rats had lower mammary mRNA abundance of xanthine oxidoreductase (XOR) and protein level of tumor necrosis factor (TNF)-α, but had higher mammary mRNA abundances of interleukin (IL)-10 and peroxisome proliferator-activated receptor (PPAR)-γ at day 14 of gestation. Following LPS infusion at day 3 postpartum, the SO-fed rats had increased plasma concentrations of FFA, C18:1n9, C18:3n3, C18:2n6 and total n-6 PUFA, higher mammary mRNA abundances of IL-1β, TNF-α and XOR but lower mammary mRNA abundance of IL-10 than the FO-fed rats.

**Conclusions:**

Mammary inflammation around parturition appeared to be attenuated by consumption of a diet rich in n-3 PUFA, which was associated with up-regulated expression of IL-10 and PPAR-γ.

## Background

Udder health is pivotal to productivity, antibiotic use and animal welfare [1]. Mastitis threatens the health of mammals all over the world including humans. For the dairy industry, mastitis is the most costly common disease, and the economic loss due to mastitis in dairy cattle is estimated at $185/cow/year in the United States [2]. For the pork industry, the infection of the mammary glands results in reduced productivity of sows and increased mortality of piglets [3]. Up to a third of lactating women will become ill because of mastitis [4]. The occurrence of mastitis is characterized by redness, swelling, and pain. However, without these symptoms, subclinical mastitis can also endanger the health of mammals, characterized by a high somatic cell count [5]. Owing to its invisible characteristic, subclinical mastitis may be neglected and bring even larger economic loss. The current method to treat mastitis is to use antibiotics [6], which may lower the quality of animal products, and threaten the health of humans. Thus, new methods dealing with clinical and subclinical mastitis are urgently needed.

It is generally considered that exogenous pathogens are the main causes of mastitis, as *E.coli* and *staphylococcus* have been confirmed to play key roles in inducing mastitis in domestic animals [6,7]. These microorganisms may activate the mammary innate immune systems and thus cause inflammatory responses. Lipopolysaccharide (LPS) has been used as the agonist in construction of mastitis models in vivo and in vitro [7,8]. Recent studies indicated that in addition to LPS, saturated fatty acids (SFA) could also activate Toll-like-receptor (TLR) 4 signaling pathway through regulation of receptor dimerization and recruitment into lipid rafts in a reactive oxygen species (ROS)-dependent manner [9]. Further studies revealed that the cooperative effect of SFA and LPS on monocytes resulted in about 3-fold higher mRNA and protein expression of pro-inflammatory cytokines than the sum of individual responses to SFA and LPS, indicating nutrient modification of TLR4–mediated inflammation [10]. It was reported that SFA were enriched in milk of mice fed western diet, and triggered ceramide accumulation and inflammation in the neonates [11]. Notably, the neonatal toxicity requires TLR but not microbiota [11], suggesting that SFA may induce inflammatory responses independent of exogenous pathogens. Studies in vivo and in vitro have demonstrated that n-3 polyunsaturated fatty acids (PUFA) could block SFA- and/or LPS-induced TLR signalling and attenuate inflammation [12]. However, little is known about whether the mammary inflammation induced by nutrients such as SFA and pathogens such as LPS could be attenuated by consumption of diets rich in n-3 PUFA.

Therefore, the objectives of the present study were to evaluate the interactive effect of diets and reproductive stages on fatty acids (FA) metabolism and inflammation as well as to determine the effect of dietary n-3 PUFA on plasma FA composition and mammary glands inflammation.

## Results

### Effect of diet type on plasma FA composition at different reproductive stages

Consumption of the SO diet increased plasma concentrations of C20:4n6 and total n-6 PUFA at day 3 postpartum (Table 1). In contrast, consumption of the FO diet increased plasma concentrations of C20:5n3 from day 14 of gestation and C22:6n3 at day 3 postpartum. Compared with the SO-fed rats, the FO-fed rats had lower plasma concentration of C16:0 at day 0 of gestation and day 3 postpartum, concentrations of C18:2n6, C20:4n6 and total n-6 PUFA at day 14 of gestation and day 3 postpartum, and concentrations of C18:0 and total SFA at the three time points evaluated, but had higher plasma concentration of C22:6n3 at day 3 postpartum, and concentrations of C20:5n3 and total n-3 PUFA at day 14 of gestation and day 3 postpartum.

**Table 1:** **Plasma FA composition (μg/mL) of rats fed different diets at different reproductive stages**^**1**^

**Item**	**Group**	**Day 0 of gestation**	**Day 14 of gestation**	**Day 3 postpartum**	**Pooled SEM**
C14:0	SO	30.69^a^	28.54^ab^	32.62^a^	13.69
	FO	14.35^b^	18.22^ab^	21.11^ab^	
C16:0	SO	822.89^a^	738.64^ab^	948.69^a^	249.21
	FO	432.50^c^	455.61^bc^	373.53^c^	
C18:0	SO	588.93^a^	526.74^a^	688.14^a^	152.56
	FO	342.09^b^	311.58^b^	239.47^b^	
C20:0	SO	21.42^a^	10.30^b^	13.32^ab^	8.91
	FO	6.33^b^	6.71^b^	4.17^b^	
SFA	SO	1463.92^a^	1304.23^a^	1682.78^a^	412.54
	FO	795.28^b^	792.12^b^	638.28^b^	
C16:1	SO	59.10^a^	47.62^ab^	57.58^a^	23.81
	FO	42.06^ab^	42.55^ab^	20.74^b^	
C18:1n7	SO	27.86^ab^	23.04^b^	42.41^a^	14.47
	FO	23.01^b^	20.98^b^	22.75^b^	
C18:1n9	SO	191.83	177.43	156.96	40.63
	FO	198.23	203.30	162.35	
C20:1	SO	30.67^ab^	26.84^abc^	40.99^a^	15.54
	FO	10.95^cd^	12.91^bcd^	5.41^d^	
MUFA	SO	309.46	274.93	297.94	86.48
	FO	274.25	279.74	211.26	
C18:3n3	SO	8.99^abc^	10.82^ab^	11.65^a^	1.84
	FO	7.07^bc^	8.42^abc^	6.43^c^	
C20:5n3	SO	7.59^b^	5.32^b^	8.00^b^	22.64
	FO	26.62^b^	87.73^a^	95.75^a^	
C22:6n3	SO	40.45^b^	37.53^b^	76.60^b^	36.39
	FO	73.89^b^	63.54^b^	146.34^a^	
n-3 PUFA	SO	64.84^c^	54.74^c^	96.25^c^	50.47
	FO	107.58^bc^	159.69^b^	248.51^a^	
C18:2n6	SO	253.41^a^	230.13^a^	262.59^a^	45.26
	FO	235.76^a^	163.29^b^	169.54^b^	
C20:4n6	SO	257.71^b^	233.28^b^	400.24^a^	87.69
	FO	246.19^b^	116.03^c^	197.67^bc^	
n-6 PUFA	SO	511.12^b^	463.41^bc^	662.83^a^	119.17
	FO	481.95^bc^	279.31^d^	367.21^cd^	
TFA	SO	2349.34^ab^	2097.31^abc^	2739.80^a^	557.06
	FO	1704.65^bc^	1510.87^c^	1465.26^c^	

^1^ Values of a certain fatty acid assigned no common superscript letter differ significantly (P < 0.05).

### Effect of reproductive stages and diet type on plasma FFA concentration and inflammation mediators in rat mammary glands

**Figure 1:**
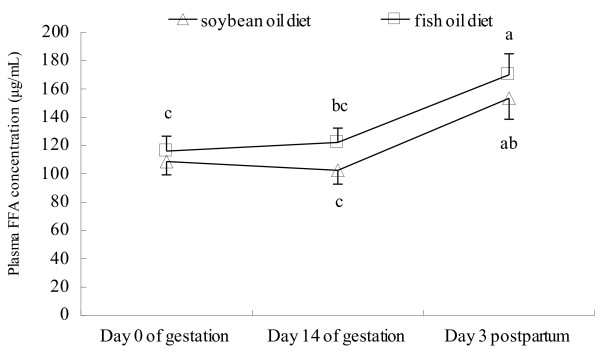
**Plasma FFA concentration of rats fed different diets at different reproductive stages.** Plasma FFA concentration was determined by ELISA using plasma collected from rats fed the SO or FO diet at day 0 of gestation, day 14 of gestation and day 3 postpartum. Statistics are shown as means ± SE. Statistics with no common letters differ significantly (P < 0.05).

**Figure 2:**
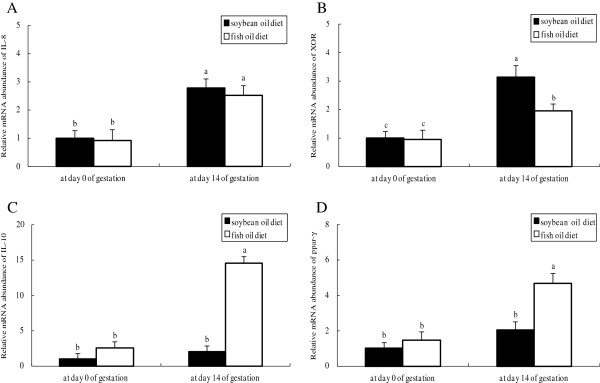
**Relative mRNA abundances of rats fed different diets at different reproductive stages.** mRNA abundances of IL-8 **(A)**, XOR **(B)**, IL-10 **(C)** and PPAR-γ **(D)** was determined by RT-PCR with mammary tissues collected from rats fed the SO or FO diet at day 0 and 14 of gestation. Statistics are shown as means ± SE. Statistics with no common letters differ significantly (P < 0.05).

**Figure 3:**
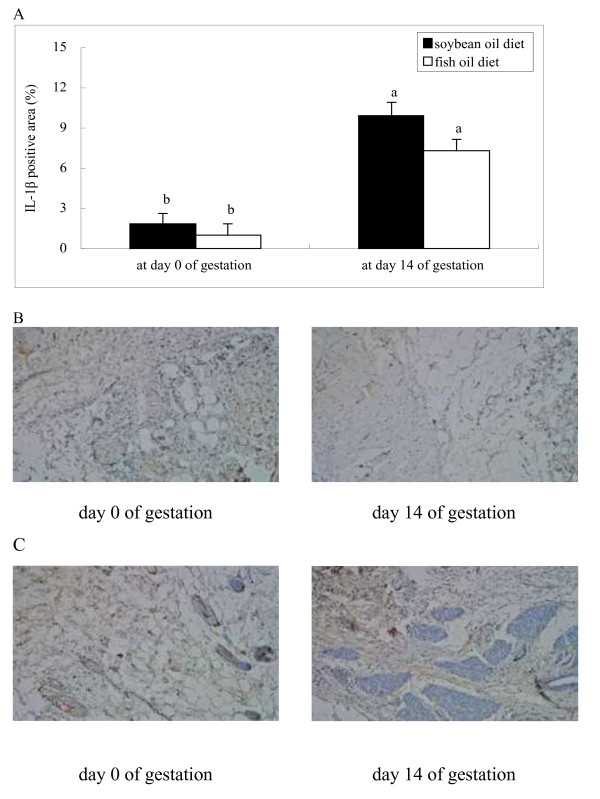
**Immunohistochemical localization of IL-1β in udder of rats fed different diets at different reproductive stages.** The microphotograph from one rat with the positive primary IL-1β antibody was visualized with DAB reaction. The area positive for IL-1β in mammary tissues of rats fed the SO diet **(B)** or FO diet **(C)** at day 0 and 14 of gestation was quantified by Easy Image 3000 software. IL-1β production is presented as the average percentage of the positively stained areas **(A)**. Statistics are shown as means ± SE. Statistics with no common letters differ significantly (P < 0.05).

**Figure 4:**
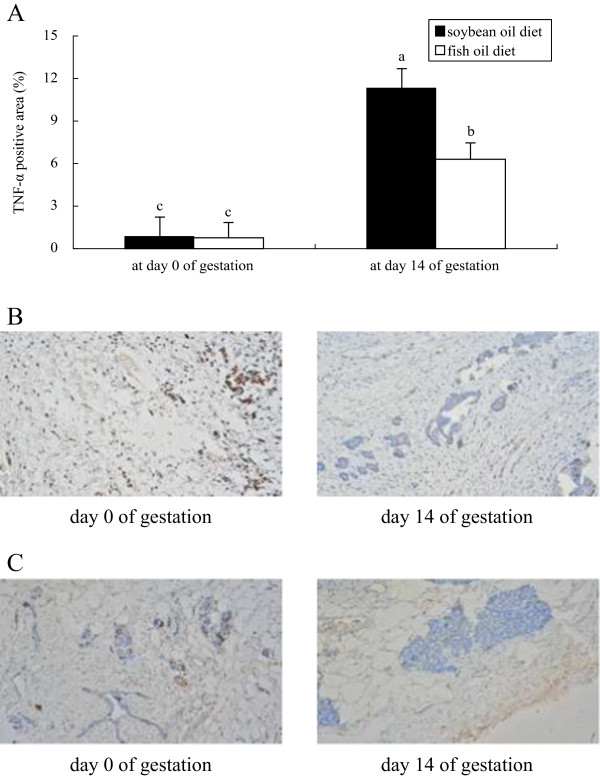
**Immunohistochemical localization of TNF-α in udder of rats fed different diets at different reproductive stages.** The microphotograph from one rat with the positive primary TNF-α antibody was visualized with DAB reaction. The area positive for TNF-α in mammary tissues of rats fed the SO diet **(B)** or FO diet **(C)** at day 0 and14 of gestation was quantified by Easy Image 3000 software. TNF-α production is presented as the average percentage of the positively stained areas **(A)**. Statistics are shown as means ± SE. Statistics with no common letters differ significantly (P < 0.05).

**Figure 5:**
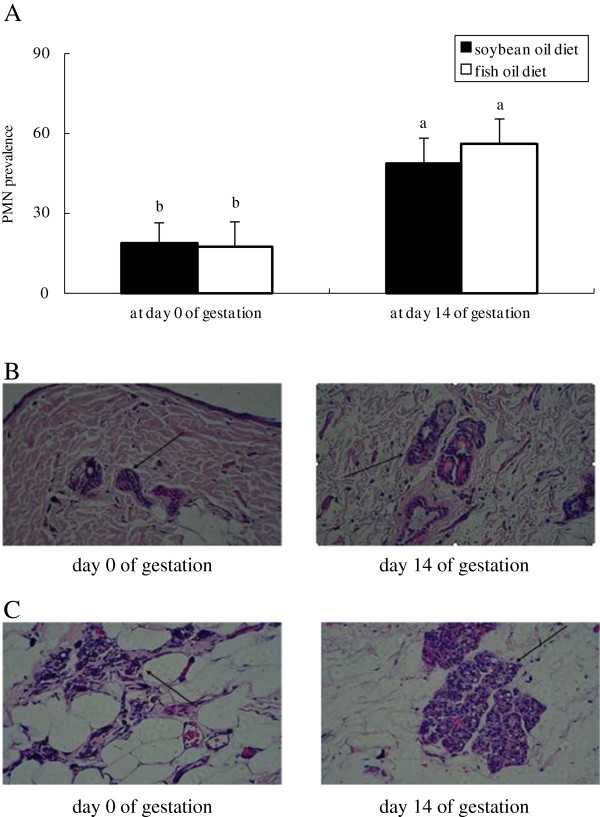
**Histopathology of mammary glands of rats fed different diets at different reproductive stages.** Hematoxylin and eosin stained slides were made with mammary tissues collected from rats fed the SO diet **(B)** or FO diet **(C)** at day 0 and 14 of gestation. PMN prevalence **(A)** in alveoli was estimated by using light microscopic (Olympus BH2, Japan) analysis at a magnification of 400×. Statistics are shown as means ± SE. Statistics with no common letters differ significantly (P < 0.05).

Plasma FFA concentration in both groups was higher at day 3 postpartum than at day 0 and 14 of gestation, with no difference observed between groups at each of the time points evaluated (Figure 1). In both groups, the mRNA abundances of IL-8 and xanthine oxidoreductase (XOR) (Figure 2), the protein levels of IL-1β (Figure 3) and TNF-α (Figure 4) as well as PMN prevalence (Figure 5) were higher at day 14 of gestation than at day 0 of gestation. Compared with the SO-fed rats, the FO-fed rats had lower mammary mRNA abundance of XOR (Figure 2) and protein level of TNF-α (Figure 4), but had higher mammary mRNA abundances of IL-10 and peroxisome proliferator-activated receptor (PPAR)-γ at day 14 of gestation (Figure 2).

### Effect of LPS infusion and diet type on plasma FFA concentration and plasma FA composition

**Figure 6:**
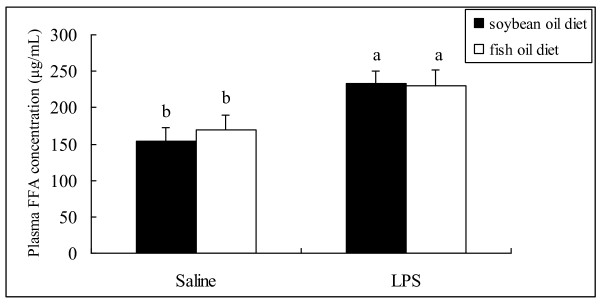
**Plasma FFA concentration of rats fed different diets and challenged with different stimulus.** Plasma FFA concentration was determined by ELISA using plasma collected from rats fed the SO or FO diet and challenged with saline or LPS. Statistics are shown as means ± SE. Statistics with no common letters differ significantly (P < 0.05).

Following LPS infusion at day 3 postpartum, both groups had increased plasma FFA concentration with no difference observed between them (Figure 6). LPS infusion resulted in increased plasma concentrations of C18:1n9, C18:3n3, C18:2n6 and total n-6 PUFA in the SO-fed rats, whereas no change was observed in the FO-fed rats. As a result, the FO-fed rats still had higher plasma concentrations of C20:5n3, C22:6n3 and total n-3 PUFA, and lower plasma concentrations of C18:2n6, C20:4n6, total n-6 PUFA, SFA and FA than the SO-fed rats following LPS infusion (Table 2).

**Table 2:** Plasma FA composition (μg/mL) of rats fed different diets and challenged with different stimulus

**Item**^**1**^	**SS**	**SL**	**FS**	**FL**	**Pooled SEM**
C14:0	32.62^ab^	45.60^a^	21.11^b^	20.19^b^	13.95
C16:0	948.69^a^	1103.10^a^	373.53^b^	581.32^b^	231.72
C18:0	688.14^a^	791.68^a^	239.47^b^	401.64^b^	138.39
C20:0	13.32^ab^	17.10^a^	4.17^c^	8.11^bc^	4.04
SFA	1682.78^a^	1957.48^a^	634.11^b^	1003.14^b^	382.43
C16:1	57.58^ab^	80.16^a^	20.74^c^	43.88^bc^	23.12
C18:1n7	42.41^ab^	51.73^a^	22.75^b^	21.87^b^	19.09
C18:1n9	156.96^b^	218.23^a^	162.35^b^	195.47^ab^	36.77
C20:1	40.99^ab^	55.48^a^	5.41^c^	21.85^bc^	18.35
MUFA	297.94^ab^	405.60^a^	211.26^b^	283.07^ab^	86.41
C18:3n3	11.65^b^	21.24^a^	6.43^b^	8.32^b^	4.67
C20:5n3	8.00^b^	11.32^b^	95.75^a^	142.18^a^	41.03
C22:6n3	76.60^c^	96.50^bc^	146.34^ab^	161.66^a^	32.93
n-3PUFA	96.25^b^	129.05^b^	248.51^a^	312.16^a^	59.83
C18:2n6	262.59^b^	379.98^a^	169.54^c^	202.36^bc^	53.52
C20:4n6	400.24^a^	485.86^a^	197.67^b^	206.75^b^	79.46
n-6PUFA	662.83^b^	865.84^a^	367.21^c^	409.11^c^	117.17
TFA	2739.80^ab^	3357.97^a^	1465.26^c^	2015.59^bc^	519.16

^abc^Values in the same row assigned no common superscript letter differ significantly (P < 0.05).

^1^SS, rats fed the SO diet and infused by saline; SL, rats fed the SO diet and infused by LPS; FS, rats fed the FO diet and infused by saline; FL, rats fed the FO diet and infused by LPS.

### Effect of diet type on inflammation mediators in LPS-infused mammary glands

**Figure 7:**
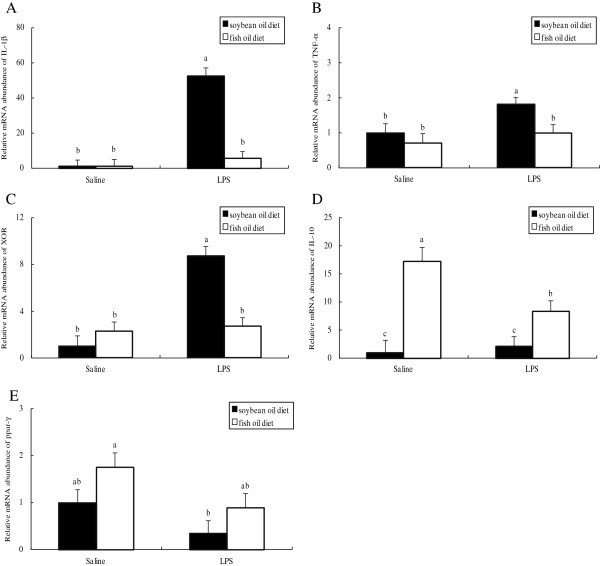
**Relative mRNA abundances of rats fed different diets and challenged with different stimulus.** mRNA abundances of IL-1β **(A)**, TNF-α **(B)**, XOR **(C)**, IL-10 **(D)** and PPAR-γ **(E)** was determined by RT-PCR with mammary tissues collected from rats fed the SO or FO diet and challenged with saline or LPS. Statistics are shown as means ± SE. Statistics with no common letters differ significantly (P < 0.05).

**Figure 8:**
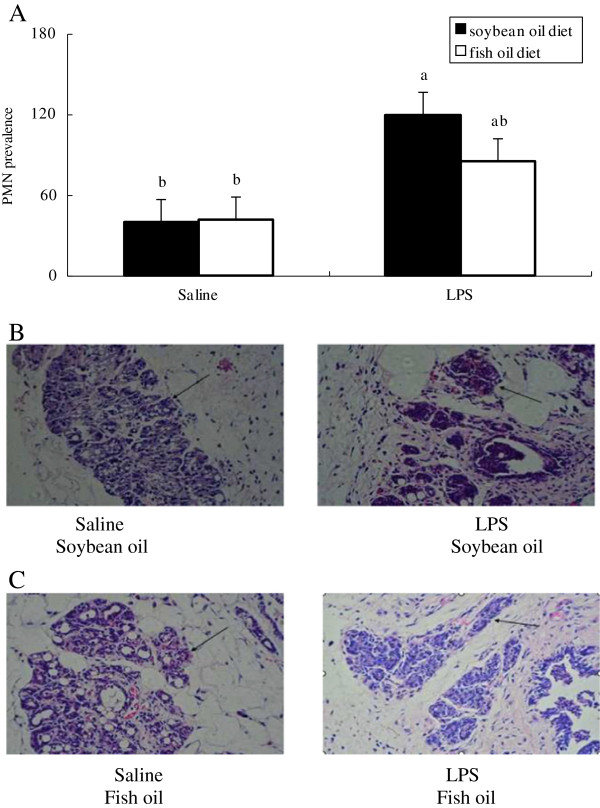
**Histopathology of mammary glands of rats fed different diets and challenged with different stimulus.** Hematoxylin and eosin stained slides were made with mammary tissues collected from rats fed the SO diet **(B)** or FO diet **(C)** and challenged with saline or LPS. PMN prevalence **(A)** in alveoli was estimated by using light microscopic (Olympus BH2, Japan) analysis at a magnification of 400×. Statistics are shown as means ± SE. Statistics with no common letters differ significantly (P < 0.05).

Mammary mRNA abundances of IL-1β, TNF-α and XOR (Figure 7) as well as PMN prevalence (Figure 8) were increased following LPS infusion at day 3 postpartum, which was observed in the SO-fed rats rather than in the FO-fed rats. Accordingly, mammary mRNA abundances of IL-1β, TNF-α and XOR following LPS infusion was lower in the FO-fed than in the SO-fed rats (Figure 7). In contrast, mammary IL-10 mRNA abundance was higher in the FO-fed than in the SO-fed rats, although it was decreased in the FO-fed rats following LPS challenge (Figure 7).

## Discussion

The beneficial effects of FO have been documented abundantly [13-15]. And evidences concerning n-3 PUFA alleviating inflammation were persuasive [16-18]. However, the effect of dietary FO on attenuating mammary inflammation has not been verified. Thus, the present study was focused on the mammary glands to test the anti-inflammatory effect of dietary FO.

Given that inflammatory responses are in strong association with FA types such as n-6 and n-3 PUFA, we firstly examined the plasma FA profile at different reproductive stages of rats receiving different diets. An amplified plasma concentration of n-3 PUFA was observed following FO consumption for 14 days in this study, which agreed well with the previous study in horses by Hall *et al*[19]. Hall *et al*[19] also found decreased plasma n-6 FA concentration in the FO-fed horses, but little variation of plasma n-6 PUFA concentration was observed in the FO-fed rats in this study. Noting that the horses used in the study of Hall *et al*[19] consumed FO for 6 weeks, a shorter consumption period (24 days) in this study may explain the little variation of n-6 PUFA. In addition, it was verified in the present study that substituting SO with FO resulted in higher plasma n-3 PUFA concentration and lower concentrations of SFA and n-6 PUFA. Amira *et al*[20] reported that decreased n-6/n-3 ratio led to higher plasma n-3 PUFA concentration and lower n-6 PUFA concentration, which was consistent with our results considering that the n-6/n-3 ratio in our experimental diets was approximately 0.5:1 in the FO diet and 10:1 in the SO diet.

Another result in the present study was that up-regulated plasma FFA emerged with the advance of gestation. As FFA have been reported to be associated with inflammation including mastitis [20,21], we further studied whether the advance of gestation was related to the expression of inflammation mediators. In both diet groups, the mRNA abundances of XOR and IL-8, protein levels of IL-1β and TNF-α as well as PMN prevalence all increased from day 0 of gestation to day 14 of gestation. TNF-α, IL-8 and IL-1β are all known as pro-inflammatory cytokines. Stimulated by a range of agents, TNF-α induces other inflammatory mediators that participated in inflammatory responses [22]. IL-8 can activate neutrophils to degranulate and induce tissue damage [23]. Moreover, IL-1β and TNF-α were elucidated to be key mediators participating in the neutrophil recruitment into the udder [24]. XOR is also an inflammatory indicator that highly expressed in mammary tissues during pregnancy and lactation [25,26]. Hence, we proposed that the advance of pregnancy was accompanied by inflammatory responses of the udder. Notably, compared with the SO-fed rats, the FO-fed rats had lower mRNA levels of XOR and TNF-α but higher mRNA levels of IL-10 and PPAR-γ, both of which are acknowledged as anti-inflammatory mediators [27,28]. The down-regulation of XOR and TNF-α may be induced by the lower SFA and n-6 PUFA concentrations and higher n-3 PUFA concentrations in plasma. Consistent with this notion, it has been shown that the decreased production of TNF was accompanied by a decreased ratio of C20:4n6 to C20:5n3 in the membrane phospholipids of mononuclear cells, which indicates the significance of systemic n-6/n-3 PUFA profile in inflammatory responses [29].

Excited by the potential effect of FO in decreasing pro-inflammatory cytokines in mammary glands, we further determined the anti-inflammatory effect of FO with a rat mastitis model. Rats at day 3 postpartum were infused with LPS or saline. LPS challenge resulted in elevated plasma FFA in both groups indicating the systemic inflammation induced by LPS. However, only in the SO-fed rats did LPS infusion stimulate the expression of IL-1β, XOR and TNF-α. During the process of mammary glands inflammation induced by advance of gestation as well as by LPS infusion, the relatively lower level of mammary pro-inflammatory mediators in the FO-fed rats may result from higher total n-3 PUFA concentration and lower SFA concentration in plasma. During lactation, the mammary blood flow increased sharply [30] and the mammary glands may become more susceptible to substances in the blood. Therefore, we assumed that the relatively higher SFA concentration in the SO-fed rats may lead to aggravated toxicity of LPS. On one hand, LPS could activate the TLR4 pathway, free NF-κB [31] and finally facilitate the expression of pro-inflammatory cytokines in the mammary glands of the SO-fed rats. On the other hand, the elevated XOR expression may enhance the generation of ROS which may participate in the TLR4 dimerization and recruitment of TLR4 into lipid rafts on condition that SFA were metabolized into ceramide [9,10]. The higher levels of PPAR-γ and IL-10 in the FO-fed rats may block the TLR4 pathway, as elucidated previously [32,33]. It has been demonstrated that DHA specifically enhanced anti-inflammatory IL-10 secretion [34]. Moreover, it has been shown in HK-2 cells that DHA and EPA can activate the mRNA expression of PPAR-γ [35]. Meanwhile, n-3 PUFA and their metabolites are natural ligands for PPAR-γ [36] and DHA for example can be metabolized by oxygenase to 17-OH and 7-OH-DHA thus facilitates PPAR-γ activation [37]. Therefore, n-3 PUFA can upregulate PPAR-γ expression and promote PPAR-γ functioning. Noticeably, maternal PPAR-γ was demonstrated to be pivotal for protecting the nursing newborns by suppressing the production of inflammatory lipids in the lactating mammary glands [38]. Additionally, it was assumed that IL-10 may inhibit the production of TNF-α and IL-6 in the mammary glands [39]. Therefore, we postulated that FO might down-regulate the mRNA expression of IL-1β, XOR and TNF-α through enhancing the expression of IL-10 and PPAR-γ.

## Conclusions

This study suggested that mammary inflammation induced by pregnancy proceeding and pathogen challenge might be attenuated by consumption of FO rich in n-3 PUFA. IL-10 and PPAR-γ appeared to be the key mediators elicited by FO consumption in ameliorating the mammary inflammation.

## Methods

### Animals, diets and treatments

All experimental protocols were approved by the Animal Care and Use Committee of Sichuan Agricultural University, and were in accordance with the National Research Council’s Guide for the Care and Use of Laboratory Animals. The experimental rats (Virgin female Sprague-Dawley rats) were purchased from Sichuan Academy of Medical Sciences-Sichuan Provincial People’s Hospital Experimental Animal Research Institute, and housed individually in metallic cages in a temperature controlled (22 ± 2°C) room with a 12 h light/dark cycle and relative humidity was maintained at 60 ± 10%.

Experimental diets (Table 3) were formulated to meet or exceed the nutrient requirements of gestating and lactating rats as recommended by AIN-93G. To create difference in inclusion levels of dietary SFA and n-3 PUFA and to make sure the two diets were isocaloric, the 7 kg fat included in the experimental diets was composed of 7 kg soybean oil (SO) in the SO diet, and 5 kg fish oil (FO), 1 kg lard and 1 kg SO in the FO diet. The FA composition of the two types of oil and experimental diets is shown in Table 4. To avoid PUFA oxidation, all diets were stored at -20 C.

**Table 3:** Ingredients and composition of experimental diets (air-dry basis)

**Ingredients**	**Content (%)**	**Composition**	**Content (%)**
Corn starch	39.75	Crude protein	16.23
Casein	20	ME, Mcal/kg	3.81
Gelatinization starch	13.2	Lysine	1.53
Sucrose	10	Methionine	0.57
Fat^1^	7	Calcium	0.50
Fiber	5	Available phosphorus	0.16
Mineral premix^2^	3.5		
Vitamin premix^3^	1		
L-cysteine	0.3		
Choline Chloride	0.25		
Total	100		

^1^The 7 kg fat was composed of 7 kg SO in the SO diet, and 5 kg FO, 1 kg lard and 1 kg SO in the FO diet.

^2^Provided per kg of diet: Calcium 5000 mg, Phosphorus 1561 mg, Potassium 3600 mg, sodium 1019 mg, Chlorine 1517 mg, magnesium 510 mg, Rion35 mg, Zinc 30 mg, Manganese 10 mg, Copper 6 mg, Selenium 0.15 mg, Iodine 0.2 mg.

^3^Provided per kg of diet: Vitamin A 4 000 IU, Vitamin D_3_ 1000 IU, Vitamin K_3_ 0.75 mg, Vitamin B_1_ 6.0 mg, Vitamin B_2_ 7.0 mg, Vitamin B_6_ 6.0 mg, Vitamin B_12_ 0.02 mg, nicotinic acid 30.0 mg, D-calcium pantothenate 15.3 mg, folic acid 2.0 mg, biotin 0.2 mg.

**Table 4:** FA composition of oil (g/100 g) and diets (g/kg) (as fed basis)

**Fatty acids**	**SO**	**FO**	**SO diet**	**FO diet**
C14:0	0.05	0.92	0.09	0.39
C16:0	10.93	9.05	5.25	5.49
C18:0	2.25	1.60	2.03	2.05
C20:0	0.02	0.12	0.20	0.07
C16:1	0.09	6.14	0.06	2.00
C18:1	27.43	23.00	11.72	10.77
C20:1	0.03	3.06	0.22	1.04
C22:1	ND^1^	2.31	0.23	0.78
C18:2n6	52.00	2.27	22.54	4.67
C18:3n3	5.80	1.49	2.18	0.68
C20:5n3	ND	21.90	0.225	4.48
C22:6n3	ND	14.60	ND	2.84
Other	1.40	13.54	0.26	2.76
∑FA ^2^	100	100	45	38
∑SFA ^3^	13.25	11.69	7.57	8.00
∑MUFA ^4^	27.55	34.51	12.23	14.58
∑PUFA ^5^	57.80	40.26	24.94	12.66
∑SFA/∑FA	13.25	11.69	16.82	21.05
∑MUFA/∑FA	27.55	34.51	27.18	38.37
∑PUFA/∑FA	57.80	40.26	55.43	33.32
∑n-3 ^6^	5.8	37.99	2.41	7.99
∑n-6 ^7^	52	2.27	22.54	4.67
∑n-6/∑n-3	8.97	0.06	9.36	0.58

^1^ND, Not detected.

^2^∑FA means the sum of content of all fatty acids evaluated.

^3^∑SFA means the sum of C14:0, C16:0, C18:0 and C20:0 content.

^4^∑MUFA means the sum of C16:1, C18:1, C20:1 and C22:1 content.

^5^∑PUFA means the sum of C18:2n6, C18:3n3, C20:5n3 and C22:6n3 content.

^6^∑n-3 means the sum of C18:3n3, C20:5n3 and C22:6n3 content.

^7^∑n-6 means the content of C18:2n6.

At the beginning of the experiment, all female rats were housed together with male rats to complete mating. When seminal plug in the vagina was detected in the morning, then that day was designated as day 0 of gestation. Forty-eight rats after mating were housed individually and began to receive the SO or FO diets. Blood and mammary tissue samples (n = 6) at day 0 and 14 of gestation and day 3 postpartum, respectively, were collected 9 hours after intramammary infusion of LPS according to the work of Miao *et al*[40]*.* The infusion was conducted according to the methods of Zhong *et al*[41]. Briefly, the inguinal mammary glands of rats were infused with 0 or 10 μg *E.coli* LPS (O55:B5, Sigma, USA) dissolved in 100 μl sterile, pyrogen-free, physiological saline. Blood samples were collected (after 12-h fast and following isoflurane anesthesia) through intra-orbital bleeding for the separation of plasma and then stored at -20°C until analysis. The fourth mammary glands were cut with scissors, and then the left and right mammary glands were snap frozen in lipid nitrogen and stored at -80°C or fixed in 4% paraformaldehyde and stored at 4°C respectively.

### FA composition analysis

Plasma FA composition was determined according to the methods described by Fernández-Real *et al*[42] with modification. In brief, 30-50 mg weighed plasma sample was mixed with four milliliter acetyl chloride and methanol solution (1:10, vol/vol). Transesterification was conducted and the pooled solvent extracts were dried by nitrogen at room temperature. The residues were dissolved in 5 ml hexane with internal standard and subjected to water bath at 80°C for 2 hours. Then 7% potassium carbonate was added, and supernatant was collected for analysis. Hewlett-Packard 6890 gas chromatograph equipped with a flame ionization detector was used to analyze the FA composition, and helium was used as carrier gas. The injector temperature was programmed at 250°C and the detector temperature was 270°C.

### FFA analysis

A commercial ELISA kit (GBD, USA) was used to determine plasma free fatty acids (FFA) concentration as described by the manufacturer’s protocols. All assays were conducted in 96-well plates and absorbance at 450 nm was detected with a microplate reader. FFA values were calculated according to the standard curve generated from the corresponding absorbance of the standard reagent.

### RNA extraction and real-time PCR

The mRNA abundances of the mammary gland samples were measured by real-time polymerase chain reaction (PCR) as previously described [43]. Total RNA was extracted using a TRIZOL Reagent kit (Invitrogen, Carlsbad, CA). The cDNA was prepared using a reverse transcription (RT) kit (TAKARA, Japan) following the manufacture’s instruction. Primers were synthesized by Chengdu Tiantai Biological Company (Chengdu, China). Beta-actin was used as an internal control according to the work of Gu et al [43]. The nucleotide primer sequences are listed in Table 5. Quantitative real-time RT-PCR analysis was performed using a 7900 real-time PCR system (Applied Biosystems, USA) and SYBR Green assays (Master Mix SYBR^®^ Green TAKARA, Japan). The specificity of PCR products were examined with melting curve analysis. Results (fold changes) were expressed as 2^-ΔΔCt^ with ΔΔCt = (Ct ij - Ct β-actin j) - (Ct i1 - Ct β-actin1), where Ct ij and Ct β-actin j are the Ct for gene i and for β-actin in a sample (named j), and where Ct i1 and Ct β-actin1 are the Ct in sample 1, expressed as the standard.

**Table 5:** PCR product sequences of oligonucleotide primers used to amplify cytokines and a house keeping gene

**Gene**		**Primer sequences(5′-3′)**	**Products size**	**Genebank accession number**
IL-1β	Forward	tgacctgttctttgaggctgac	113 bp	M98820.1
	Reverse	cgagatgctgctgtgagatttg		
TNF-α	Forward	ccactctgacccctttactctga	154 bp	NM_013693.2
	Reverse	ctgtcccagcatcttgtgtttc		
IL-8	Forward	ccagcaggaaaccagaagaaag	123 bp	NM_001173399.2
	Reverse	caactttgtcacgaccataccc		
IL-10	Forward	gctggacaacatactgctgaca	112 bp	NM_012854.2
	Reverse	ctggggcatcacttctaccag		
PPAR-γ	Forward	gccctttggtgactttatggag	170 bp	NM_013124.3
	Reverse	gcagcaggttgtcttggatgt		
XOR	Forward	gattctcacacacctcctgacg	156 bp	NM_011723.2
	Reverse	ccccacacacacacacacactat		
β-actin	Forward	ctgtgtggattggtggctctatc	133 bp	NM_031144.2
	Reverse	gctcagtaacagtccgcctagaa		

### Histopathologic examination

Mammary tissue samples fixed in 4% paraformaldehyde for 24 h were further processed with standard dehydration and paraffin-wax embedding procedures to produce tissue blocks. Hematoxylin and eosin stained slides were made as described previously [40]. The prevalence of polymorphonuclear neutrophils (PMN) in alveoli was estimated by using light microscopic (Olympus BH2, Japan) analyses at a magnification of 400× as previously described [40]. Briefly, four sections of mammary tissues were chosen for each rat. Ten fields were randomly selected per sample. Results were presented as average PMN infiltration scores for each time point.

### Immunohistochemistry

Polyclonal antibodies combined with the avidin-biotinperoxidase complex (ABC) technique were used for the immunohistochemical detection of interleukin (IL)-1β (Abnova, USA) and tumor necrosis factor (TNF)-α (Novus, USA). All samples from one animal were analyzed within the same assay run, and within each assay run treatment animals to be compared were included. The quantification of IL-1β and TNF-α protein expression in mammary tissue samples was performed as described [44]. For each sample, a relative value of the amount of cytokine produced was expressed as the average percentage of the positively stained areas.

### Statistic analysis

All statistical evaluation was performed by using the General Linear Model procedures of SAS statistical package (V8.1, SAS Institute Inc., Cary, NC). The statistic model used is as follows: *Y*_*ijk*_ = *μ* + *A*_*i*_ + *B*_*j*_ + (*A* × *B*)_*ij*_ + *ϵ*_*ijk*_, where *Y* is the analysed variable, *μ* the overall mean, *A* the effect of diet, *B* the effect of time or LPS, *A* × *B* the effect of diet × time or LPS interaction, and *ϵ* the random error. Least-squares means comparison was used to evaluate differences among treatments. *P* values ≤ 0.05 were considered statistical significance.

## Competing interests

The authors declare that they have no competing interests.

## Authors’ contributions

ZF, DW, DC and KZ designed the research; JH, FX, SL, XZ, LC, YL, SX, GT, QZ and BY performed the research; SL, ZF, JH and FX analyzed the data and wrote the paper. All authors read and approved the final manuscript.
